# New Appliances and Things Medical

**Published:** 1907-05-11

**Authors:** 


### NEW APPLIANCES AND THINGS MEDICAL.

The Electric Hygienic Food Warmer is a most ingeniously simple contrivance for warming infant and invalid food. It consists essentially of a high power electric light enclosed in a reflecting chamber beneath a perforated zinc plate, on which the bottle containing the food rests. The whole is covered with a shawl or large napkin, the plug of the wire is fitted to an ordinary electric light holder, and the current is turned on. The result is a hot air heater, simple, perfectly safe, and, as we have convinced ourselves, relatively cheap. There is no smell, no risk of burning, no uncleanliness, and no chance of "overboiling" or spoiling the food. The heater is supplied by Messrs. Reavell, Bros. and Company of Bondgate, Alnwick from whom catalogues and full particulars may be obtained.

